# Hydroxyurea Could Be a Good Clinically Relevant Iron Chelator

**DOI:** 10.1371/journal.pone.0082928

**Published:** 2013-12-09

**Authors:** Khushnooma Italia, Roshan Colah, Kanjaksha Ghosh

**Affiliations:** 1 Hematogenetics Department, National Institute of Immunohaematology, I.C.M.R., Mumbai, Maharashtra, India; 2 National Institute of Immunohaematology, I.C.M.R., Mumbai, Maharashtra, India; CINVESTAV-IPN, Mexico

## Abstract

Our previous study showed a reduction in serum ferritin of β-thalassemia patients on hydroxyurea therapy. Here we aimed to evaluate the efficacy of hydroxyurea alone and in combination with most widely used iron chelators like deferiprone and deferasirox for reducing iron from experimentally iron overloaded mice. 70 BALB/c mice received intraperitonial injections of iron-sucrose. The mice were then divided into 8 groups and were orally given hydroxyurea, deferiprone or deferasirox alone and their combinations for 4 months. CBC, serum-ferritin, TBARS, sTfr and hepcidin were evaluated before and after iron overload and subsequently after 4 months of drug therapy. All animals were then killed. Iron staining of the heart and liver tissue was done using Perl’s Prussian Blue stain. Dry weight of iron in the heart and liver was determined by atomic absorption spectrometry. Increased serum-ferritin, TBARS, hepcidin and dry weight of iron in the liver and heart showed a significant reduction in groups treated with iron chelators with maximum reduction in the group treated with a combination of deferiprone, deferasirox and hydroxyurea. Thus hydroxyurea proves its role in reducing iron from iron overloaded mice. The iron chelating effect of these drugs can also be increased if given in combination.

## Introduction

Chronic blood transfusion therapy in β-thalassemia leads to an average iron accumulation of approximately 0.3-0.5mg/kg/day [1]. Treatment of β-thalassemia is expensive with the life-time cost estimated to be US$1,10,000/-. In developing countries like India, sufficient safe blood is not readily available and the cost of iron chelation is prohibitive for most families leading to severe anemia and death [2,3].

Pharmacological induction of HbF has been a major therapeutic approach to hemoglobinopathies. Our earlier studies with hydroxyurea therapy showed a significant improvement in the clinical condition in 98% of sickle cell anemia patients and 60% of the β-thalassemia intermedia patients [4,5]. This was in agreement with many studies [6-8]. Also noticed was a sharp reduction in the serum ferritin of some patients on hydroxyurea therapy without changing the chelation regimen [5,9,10]. However, it was not possible in this clinical setup to conclusively conclude on clinically significant iron removal effect of hydroxyurea as most of the patients were on regular blood transfusion and reduction in serum ferritin in these patients could have been due to reduction in the number of blood transfusions. 

Hence we designed an animal study using iron overloaded Balb/c mice where hydroxyurea alone and in combination with other clinically used iron chelators were administered to evaluate the ability of hydroxyurea to function as an independent iron chelator and synergistic or antagonistic effect of this drug when combined with other clinically used iron chelators at therapeutic dose. 

## Materials and Methods

Blood from 70 Balb/c mice of either sex (6–8 weeks old) was collected in EDTA and plain vials for a complete blood count (CBC) (Sysmex-K1000 Hematology Counter), estimation of serum ferritin levels using the mouse ferritin ELISA kit (Immunology Consultants Laboratory, Inc. Newberg, OR), Thiobarbituric Acid Reactive Substances (TBARS), serum transferrin receptor (sTfr) and hepcidin by ELISA (Shanghai BlueGene Biotech Co. Ltd, Shanghai, China). All the mice received intraperitonial injections of iron sucrose at 5 mg/week every alternate day for 12 weeks. The mice were given regular diet. However, at this stage we lost 6 of the 70 mice due to iron toxicity. Blood was collected for serum ferritin after giving iron to assess iron overloading till the serum ferritin levels reached around 17000 ng/ml (normal level around 800 ng/ml)

After iron loading a rest period of 20 days was given to all the mice. They were then divided into 8 groups each comprising of 8 mice and were orally given deferiprone, hydroxyurea or deferasirox individually and in combinations for 4 months daily as shown below at a therapeutically adjusted dose for mice; 

Group-1 - Control-no medication was given after iron loading.

Group-2 - 75mg/kg/day deferiprone (Kelfer, Cipla, India).

Group-3 - 20mg/kg/day hydroxyurea (Cipla, India).

Group-4 - 20mg/kg/day deferasirox (Asunra, Novartis Pharma, India).

Group-5 - 20mg/kg/day hydroxyurea + 75mg/kg/day deferiprone.

Group-6 - 20mg/kg/day hydroxyurea + 20mg/kg/day deferasirox.

Group-7 - 20mg/kg/day deferasirox + 75mg/kg/day deferiprone.

Group-8 - 20mg/kg/day hydroxyurea + 20mg/kg/day deferasirox + 75mg/kg/day deferiprone. 

After 4 months, all the mice were killed and blood was collected for CBC, serum ferritin, TBARS, serum transferrin receptor (sTfr) and hepcidin. Tissue iron staining of the heart and liver tissue using standard Perl’s Prussian blue stain, hematoxylin and eosin stain (H & E stain) and Reticulin stain were done and graded. A section of the heart and the liver biopsy were evaluated for iron/unit μg/gm of tissue by atomic absorption spectrophotometry (AA-6300 Shimadzu) in the iron overloaded mice as compared to mice that were not given iron. The study was cleared by the animal ethics committee of our Institute (ICMR/NIIH/AnimalHouse/Project-1/2011-2012). 

### Statistical Analysis

Student’s t-test was used to compare the findings between different groups. Data was presented as mean + SD. SYSTAT 10 software and Microsoft Excel 2000 was used for all statistical analysis. 

## Results

The baseline serum ferritin of the 70 balb/c mice was 815.5 + 222.4 ng/ml. This level was increased to 16272.7 + 4480.3 ng/ml after intraperitonial injection of iron sucrose for 12 weeks. Table 1 show the results of the serum ferritin levels seen before and 4 months after administration of deferiprone, deferasirox and hydroxyurea alone as well as in combination of hydroxyurea with the other two iron chelators in the iron overloaded mice.

**Table 1:** Serum ferritin, TBARS assay, serum transferrin receptor and hepcidin levels of mice in the 8 groups.

	**Serum ferritin (ng/ml)**		**TBARS assay (µM/L)**		**Serum Transferrin Receptor (sTfr) (ng/ml)**		**Hepcidin (ng/ml)**	
**Normal levels**	815 ± 222		1.6 ± 1.0		9.4 ± 1.8		47.3 ± 6.3	
	Before	After (End of study)	Before	After (End of study)	Before	After (End of study)	Before	After (End of study
**Iron overloaded mice (Group 1)**	17100 ± 3414	9565 ± 768*	6.5 ± 2.5	6.0 ± 2.0	5.9 ± 2.2	6.8 ± 2.1	123.5 ± 15.5	123.5 ± 15.6
**Iron overloaded and deferiprone treated mice (Group 2)**	15500 ± 3505	5200 ± 1165*^#^	6.2 ± 2.7	3.1 ± 1.8*	5.3 ± 2.0	6.6 ± 2.2	125.1 ± 17.6	118.7 ± 25.3
**Iron overloaded and hydroxyurea treated mice (Group 3)**	17125 ± 2799	5350 ± 1205*^#^	5.9 ± 3.0	3.2 ± 1.9*	4.6 ± 2.4	5.7 ± 2.0	120.1 ± 13.9	107.2 ± 14.1
**Iron overloaded and deferasirox treated mice (Group 4)**	15625 ± 3113	5162 ± 2257*^#^	6.8 ± 2.8	3.7 ± 1.9*	4.0 ± 2.7	4.9 ± 2.3	127.1 ± 12.7	103.1 ± 15.2*
**Iron overloaded and deferiprone and hydroxyurea treated mice (Group 5)**	16333 ± 4274	5785 ± 388*^#^	7.0 ± 2.0	2.5 ± 2.0*	5.5 ± 2.1	6.7 ± 2.0	120.5 ± 13.9	105.6 ± 9.3*
**Iron overloaded and hydroxyurea and deferasirox treated mice (Group 6)**	16187 ± 3081	5100 ± 1701*^#^	6.5 ± 2.7	3.3 ± 1.7*	3.6 ± 2.7	5.1 ± 2.8	122.8 ± 14.10	88.6 ± 10.6*
**Iron overloaded and deferiprone and deferasirox treated mice (Group 7)**	18285 ± 3401	5729 ± 660*^#^	6.9 ± 3.0	3.0 ± 2.0*	3.1 ± 0.6	4.7 ± 1.0	128.5 ± 12.8	105.2 ± 23.8*
**Iron overloaded and deferiprone, deferasirox and hydroxyurea treated mice (Group 8)**	15833 ± 2857	2515 ± 409*^#^	6.9 ± 2.7	2.2 ± 1.8*	3.2 ± 1.5	4.2 ± 1.9	122.7 ± 17.3	87.0 ± 7.9*

* statistically significant reduction (p<0.001) before and 4 months after treatment.

^#^ statistically significant reduction (p<0.001) in serum ferritin levels compared between serum ferritin level after 4 months in group 1 and serum ferritin levels after 4 months in group 2, 3, 4, 5, 6, 7, 8.

A statistically and clinically significant reduction in mean serum ferritin levels (p<0.001) was observed in group 3, given hydroxyurea alone and also in the other groups (2,4–8) with maximum reduction seen in group 8 where all three drugs were given in combination (Table 1). However, when the serum ferritin levels of the group which did not receive any treatment for 4 months were compared with the groups which received treatment, a statistically significant reduction (p<0.001) was observed in all the groups receiving treatment with deferiprone, hydroxyurea or deferasirox alone or in combination. 

The mean MCV of the mice in the hydroxyurea treated group showed an increase from 40.2 + 1.5 to 42.5 + 2.3 fl, however none presented with neutropenia. The TBARS assay done using mice plasma before intraperitonial injection of iron sucrose showed a concentration of 1.6 + 1.07 μM/L. This concentration of TBARS increased to 6.5 + 2.5 μM/L after intraperitonial injection of iron sucrose. Table 1 shows the results of the reduction in mean TBARS levels seen after administration of deferiprone, deferasirox and hydroxyurea as well as a combination of hydroxyurea with the other iron chelators for 4 months. TBARS levels did not decrease after 4 months in group 1 where the mice were not given any treatment. A statistically significant reduction (p<0.001) in mean TBARS levels was seen in mice in group 2, 3, 4, 5, 6, 7 and 8 after treatment with the respective drugs with maximum reduction seen in mice from group 5 and 8. 

A statistically significant decrease in mean sTfr levels was seen in comparison to normal mice due to iron overloading which increased after the mice received different drugs (not statistically significant), however normal levels were not reached as the complete removal of iron was not achieved (Table 1). The mean hepcidin levels also increased on iron overloading as expected. After treatment, these levels significantly reduced in groups 4, 5, 6, 7 with the maximum reduction in group 8, while the reduction in group 2 and 3 was not statistically significant (Table 1).

On comparing the dry weight of iron levels/gm of liver and heart tissues in different groups against that which were not treated with any drug, it was observed that the mean dry weight of liver iron showed a significant reduction (p<0.001) in groups 5, 6, 7 with the maximum reduction in group 8 as compared to group 1 not treated with any drug. A statistically significant reduction (p<0.001) was also seen in the mean dry weight of heart iron in groups 2, 3, 4, 5, 6, 7 and 8 as compared to group 1 (Table 2).

**Table 2:** Dry weight of iron from the liver and the heart tissues of mice.

	**Dry weight of Iron (µg/gm)**	
	Liver tissue	Heart tissue
**Normal levels**	101.3 ± 19.1	57.2 ± 41.6
**Iron overloaded mice (Group 1)**	11945 ± 2253	389 ± 188
**Iron overloaded and deferiprone treated mice (Group 2)**	9319 ± 2865	138 ± 66*
**Iron overloaded and hydroxyurea treated mice (Group 3)**	10316 ± 2820	150 ± 128*
**Iron overloaded and deferasirox treated mice (Group 4)**	11135 ± 1878	171 ± 98*
**Iron overloaded and deferiprone and hydroxyurea treated mice (Group 5)**	7136 ± 1631*	208 ± 200*
**Iron overloaded and hydroxyurea and deferasirox treated mice (Group 6)**	7397 ± 1570*	159 ± 141*
**Iron overloaded and deferiprone and deferasirox treated mice (Group 7)**	6339 ± 934*	97 ± 69*
**Iron overloaded and deferiprone, deferasirox and hydroxyurea treated mice (Group 8)**	6739 ± 1764*	89 ± 60*

* Statistically significant reduction (p<0.001) in dry weight of liver or heart iron of the particular group compared to the group 1

The Perl’s Prussian blue staining showed the presence of high (5+) iron content in the iron overloaded liver and heart of mice, which after treatment reduced (2 to 3+) in groups 2, 3 and 4 and further reduced (1 to 2+) in groups 5, 6, 7 and 8. The Reticulin staining of the tissues showed no fibrosis.

## Discussion

This study of evaluating the role of hydroxyurea, as a clinically relevant iron chelator in removing iron from iron overloaded mice is an extension of our previous study on hydroxyurea therapy among patients with different hemoglobinopathies [4,5,11]. Our earlier work had shown that the high serum ferritin levels seen in sickle cell anemia patients [mean 1329 ± 700 ng/μl] decreased to 722 ± 578 ng/μl after two years of hydroxyurea therapy [4]. A statistically significant reduction in mean serum ferritin levels was also seen in β-thalassemia intermedia, β-thalassemia major and HbE-β-thalassemia patients after hydroxyurea therapy [5,11]. Another study from Iran also showed reduction in mean serum ferritin levels after one year of hydroxyurea therapy from 2751.44 ng/mL to 1594.20 ng/mL (p<0.001) in 49 β-thalassemia patients [10].

In our present study age matched Balb/c mice were overloaded with iron and then treated with different drugs. This mode of iron administration closely mimics the route of transfusional iron overload seen in β-thalassemia patients. The dose of iron administered to the mice was equivalent to the iron accumulated over a period of 10 years of blood transfusions in a β-thalassemia patient without chelation [12]. This helped in maintaining a check on the iron supplied to the body of the mice which was a challenge in case of a continuously transfused β-thalassemia patient. 

The two iron chelators deferiprone and deferasirox are the ones widely used in India as iron chelators and contribute the maximum to the cost of management of a thalassemic child [3]. The 4 months treatment given to the mice with different drugs alone and in combination was equivalent to around 13 years of treatment in humans. 

The experiment was designed to cover most aspects of iron intake and metabolites in the body. Serum ferritin, a standard test for estimation of iron in the body is sometimes falsely increased due to infection or inflammation. This test was further supported by Perl’s Prussian blue staining technique and the dry weight of liver and heart tissue iron. These invasive methods of iron estimation though not recommended in modern times, T2* MRI does the same with precision and without invasion in humans [13,14]. 

The decrease in serum ferritin in the mice in group 1 mice, who were not treated after iron loading showed the ability of the mouse body to remove iron without any chelaton after 4 months. This ability if compared with the human body will take very long to remove iron from the body which is not possible to monitor in a continuously transfused patient. 

The TBARS assay helps to measure the oxidative stress in the cellular environment resulting in the formation of highly reactive and unstable lipid hydroperoxides due to iron overload. There was an increase in the TBARS levels after administration of intraperitonial injections of iron sucrose. The different levels of TBARS using different chelation also show deferiprone and hydroxyurea are equivalent in suppressing oxidant damage (TBARS) caused by iron overload. Combination of deferiprone and hydroxyurea has a synergistic effect in reducing TBARS but deferasirox does not significantly contribute to this effect. Hepcidin levels were also increased indicating decreased iron absorbance from food due to iron overload in the body [15]. The estimation of transferrin receptor and hepcidin further proved our hypothesis and showed that hydroxyurea can by itself, or in combination with other widely used iron chelators remove iron from the body of iron overloaded mice. 

A statistically significant decrease in the mean dry weight of the liver and heart iron was seen after treatment. The maximum reduction seen in heart tissue iron concentration in group 8 where all three drugs were given in combination to the iron overloaded mice suggests the easy accessibility and early removal of the iron stored in cardiac tissues by a combination of all the three drugs. This may be of great importance as cardiac siderosis is the leading cause of death in patients with transfusion-dependent β-thalassemia [14].

Thus, taking into consideration the different parameters studied in iron overloaded mice, it was seen that hydroxyurea treatment alone also showed an iron chelation effect. This may be attributed to the production of nitric oxide in the metabolism of hydroxyurea which appears to be involved in cellular defense against the iron-mediated reactive oxygen species generation by inducing iron removal from cells [16,17]. Kayyali et al. 1996 also demonstrated hydroxyurea to be an iron chelator. Hydroxyurea is considered as a radical scavenger and contains the hydroxamate function and therefore is also an iron chelator [18]. 

Recently, the increase in the use of hydroxyurea in hemoglobinopathies has added to the cost of management [4,5,11,19,20]. However, if hydroxyurea provides an additional benefit of iron chelation, it will be cost effective in the long run. This is the first study which proves the role of hydroxyurea in removing the labile iron from the iron overloaded mice along with reduction of oxidative stress due to iron overload. It also shows that hydroxyurea when used in combination with the other two iron chelators shows the maximum chelation effect. This could be helpful in optimum removal of iron from the body. 
